# Enhancement of recombinant myricetin on the radiosensitivity of lung cancer A549 and H1299 cells

**DOI:** 10.1186/1746-1596-9-68

**Published:** 2014-03-20

**Authors:** Shijie Zhang, Lei Wang, Hongchun Liu, Guoqiang Zhao, Liang Ming

**Affiliations:** 1 Department of Clinical Laboratory, The First Affiliated Hospital of Zhengzhou University, No.1, Jianshe Road, Zhengzhou 450052, PR China; 2 Department of Emergency, The First Affiliated Hospital of Zhengzhou University, No.1, Jianshe Road, Zhengzhou 450052, PR China; 3 College of Basic Medical Sciences, Zhengzhou University, No.100, Kexue Avenue, Zhengzhou 450001, PR China

**Keywords:** Myricetin, Pulmonary carcinoma, Radiosensitizer, Radiotherapy

## Abstract

**Objective:**

Myricetin, a common dietary flavonoid is widely distributed in fruits and vegetables, and is used as a health food supplement based on its immune function, anti-oxidation, anti-tumor, and anti-inflammatory properties. The aim of this study was to investigate the effects of myricetin on combination with radiotherapy enhance radiosensitivity of lung cancer A549 and H1299 cells.

**Methods:**

A549 cells and H1299 cells were exposed to X-ray with or without myricetin treatment. Colony formation assays, CCK-8 assay, flow cytometry and Caspase-3 level detection were used to evaluate the radiosensitization activity of myricetin on cell proliferation and apoptosis in vitro. Nude mouse tumor xenograft model was built to assessed radiosensitization effect of myricetin in vivo.

**Results:**

Compared with the exposed group without myricetin treatment, the groups treated with myricetin showed significantly suppressed cell surviving fraction and proliferation, increased the cell apoptosis and increased Caspase-3 protein expression after X-ray exposure in vitro. And in vivo assay, growth speed of tumor xenografts was significantly decreased in irradiated mice treated with myricetin.

**Conclusions:**

The study demonstrated both in vitro and in vivo evidence that combination of myricetin with radiotherapy can enhance tumor radiosensitivity of pulmonary carcinoma A549 and H1299 cells, and myricetin could be a potential radiosensitizer for lung cancer therapy.

**Virtual slides:**

The virtual slide(s) for this article can be found here: http://www.diagnosticpathology.diagnomx.eu/vs/5791518001210633

## Introduction

The morbidity and mortality of lung cancer take the first place among malignant tumors in the world [1]. Surgery, chemotherapy and radiation are the most three major therapeutic options for treatment of lung cancer. In particular, more than 50% of newly diagnosed lung cancer patients worldwide receive radiotherapy (alone or in combination with chemotherapy or surgery) at some point during their treatment [2]. However, the curative potential of radiotherapy is often limited by intrinsic radioresistance of cancer cells and systemic dose-limiting normal tissue toxicity [3-5]. Therefore, there is increasing interest in enhancing the radiosensitivity of lung cancer cells for development of effective therapies and improve patient survival with the added benefit of reduced systemic toxicity.

Recently, much radiosensitization approaches aim to target DNA damage responses (DDR) because the overall function of DDR is to promote cellular survival [6-8]. And some drugs had been reported to used as radiosensitizers targeting DDR [9-11]. However their activities in vivo had not been fully characterized. Though promising, one of the major concerns of these radiosensitizers was that they might increase normal tissue damage after radiotherapy if used systematically. Therefore, It was essential to find radiosensitizers, which could lower the radiation dose-response threshold for cancer cells without enhancing the radiosensitivity of normal cells [12,13].

Currently, there is growing interest in the therapeutic applications of bioflavonoids for the treatment and prevention of diseases in humans. Myricetin (3, 3’ ,4’ , 5, 5’ , 7- hexahydroxyflavone) is a naturally occurring flavonoid that is commonly found in tea, berries, fruits, vegetables, and medicinal herbs *et al.*[14,15]. Previous studies have shown that myricetin has antioxidant, anti-inflammatory and potent anticancer effects [15-18]. In addition, some studies have also shown that myricetin and quercetin (the congener of myricetin) can reduce UV radiation-induced skin damage and increase white blood cells in X-ray irradiated mice and human peripheral blood lymphocytes after radiotherapy [18-21]. Recently, there was a study show that combination of quercetin with radiotherapy could enhance tumor radiosensitivity [22]. It was interesting to investigate whether myricetin has the function in enhancing the radiosensitivity of cancer cells.

Non-small cell lung cancer (NSCLC) is the most predominant type of lung cancer, and about 70-80% of lung cancer fall under the classification of NSCLC with adenocarcinoma as the most common subtype [23]. In this study, we engaged NSCLC cell A549 and H1299 to observe the effects of myricetin on combination with radiotherapy enhances tumor radiosensitivity in vitro and in vivo, in order to provided a novel insight into myricetin as a potential agent for lung cancer radiosensitizers.

## Materials and methods

### Cell lines and cell culture

The human lung cancer cell line A549 and H1299 were purchased from Shanghai Institutes for Biological Sciences, Chinese Academy of Sciences. The cells were routinely cultured in DMEM (Gibco, USA) supplemented with 10% heat-inactivated fetal bovine serum (FBS), 100 U/mL penicillin and 100 μg/mL streptomycin in a humidified cell incubator with an atmosphere of 5% CO_2_ at 37°C.

### Experimental treatment

Experimental irradiation (IR) was performed at a dose rate of 2.0 Gy/min in an X-ray linear accelerator (Elekta Precise, Stockholm, Sweden) at room temperature. Myricetin (>95% purity) was purchased from Sigma-Aldrich. Treatment was given for 24 hours prior to ionizing radiotherapy or sham radiotherapy. In vitro cell assay, myricetin was added into the culture (final concentration 25 μM) at 1 h before irradiation.

### Colony-forming survival assay

The overall survival of the cells treated with myricetin or irradiation alone or in combination, was assessed by the rate of colony formation. The A549 and H1299 cells were irradiated, and the dose rate was 2.0 Gy/min. Cells were plated into 6-well plates and exposed to doses of 0, 2, 4, 6 and 8 Gy radiation, while myricetin (final concentration 25 μM) was added into the culture at 1 h before IR and maintained for 24 h. 24 hours after radiation, all cells were then washed, trypsinized, counted, and plated into 10 cm dishes containing DMEM supplemented with 10% FBS until colony formation was visible, which usually occurred in approximately 2 weeks. The colonies formed were stained with crystal violet, and the colonies with >50 cells scored as surviving colonies. The plating efficiency was calculated by dividing the average number of colonies per dish by the amount of cells plated. Survival fractions were calculated by normalization to the plating efficiency of appropriate control groups.

### In vitro cell proliferation assay

A549 and H1299 cell lines in logarithmic phase growth were seeded into 96-well plates at a density of 1 × 10^4^ cells/well with three replicate wells of each four groups (control group, radiotherapy (dose rate of 2 Gy) alone group, myricetin (25 μM) group and combination group). 24, 48 and 72 hours after irradiation, OD was measured by WST (water-soluble tetrazolium salt) assay using microplate computer software (Bio-Rad Laboratories, USA) according to the protocol of Cell Counting Kit-8 (CCK8) assay kit (Dojindo, Japan). 450 nm absorbance (A450) was read on a microplate reader (168–1000 Model 680, Bio-Rad, Hercules, USA). The curves of cell proliferation were plotted. The experiments were performed in triplicate.

### Cell apoptosis assay

A549 and H1299 cell lines in logarithmic phase growth were seeded in 6-well plate with 2.0 × 10^5^ cells in each well. Four groups (control group, radiotherapy alone group, myricetin group, and combination group) cells were harvested and counted at 24 hours after irradiation. The tests were performed using the annexin V-FITC/PI apoptosis detection kit. The cell pellets were resuspended in 195 μL of binding buffer and stained with 5 μL each of annexin V-FITC and PI staining solution for 10 minutes at room temperature in the dark. Flow cytometry was performed with the FACScan system using CellQuest software. Cell apoptosis rate was calculated as: (the number of cell apoptosis in each group/the total number of cells in each group) × 100%.

### Western blot analysis

Cell harvest was same as the method of cell apoptosis assay. Total protein was extracted from each group cells using RIPA buffer containing PMSF. A BCA protein assay kit (Beyotime, Haimen, China) was used to determine total protein concentration. Proteins were electrophoresed by SDS-PAGE and transferred onto PVDF membranes. After blocking, the membranes were incubated overnight at 4°C with diluted primary antibody (rabbit anti-caspase 3 antibody, 1:1000, Invitrogen) and followed by incubation with an HRP-conjugated secondary antibody (Santa Cruz Biotechnology). An antibody against beta-actin(Santa Cruz Biotechnology) served as an endogenous reference. Protein intensity were scanned on Typhoon PhosphorImager (GE Healthcare) for fluorescent signal. Experiments were performed in triplicate.

### Human tumor xenograft model in nude mouse

Immunodeficient female BALB/C nude mice, 5-6 weeks old, were from the Experimental Animal Center of Henan province, China. The nice were subcutaneously injected in the dorsal scapular region with A549 cells. In addtion, a sufficient number of mice were implanted in order that tumors in a weight range as narrow as possible were selected for the trial on the day of treatment initiation (Days 15 after tumor cells implantation). The tumors were allowed to reach about 150 mm^3^ in size before the start of treatment. Mice in the different groups were treated intraperitoneally with myricetin alone (20 mg/kg, once daily for 12 days) or myricetin (1 h before radiation) plus irradiation at 2 Gy fractions to a total dose of 20 Gy. The tumor volume were measured with a caliper every 5 days, and tumor volume was calculated using the formula: volume = π(length × width^2^)/6. This study was carried out in strict accordance with the recommendations in the Guide for the Care and Use of Laboratory Animals of Zhengzhou University. The protocol was approved by the Committee on the Ethics of Animal Experiments of Zhengzhou University. All surgery was performed under sodium pentobarbital anesthesia, and all efforts were made to minimize suffering.

### Statistical analysis

SPSS 13.0 was used for statistical analysis. One-way analysis of variance (ANOVA) was used to analyze the significance between groups. Multiple comparisons were made using the Least Significant Difference test when the probability for ANOVA was statistically significant. All data represent mean ± SD. Statistical significance was set at *P* < 0.05.

## Results

### Myricetin increased radiosensitization of tumor cells in vitro

**Figure 1:**
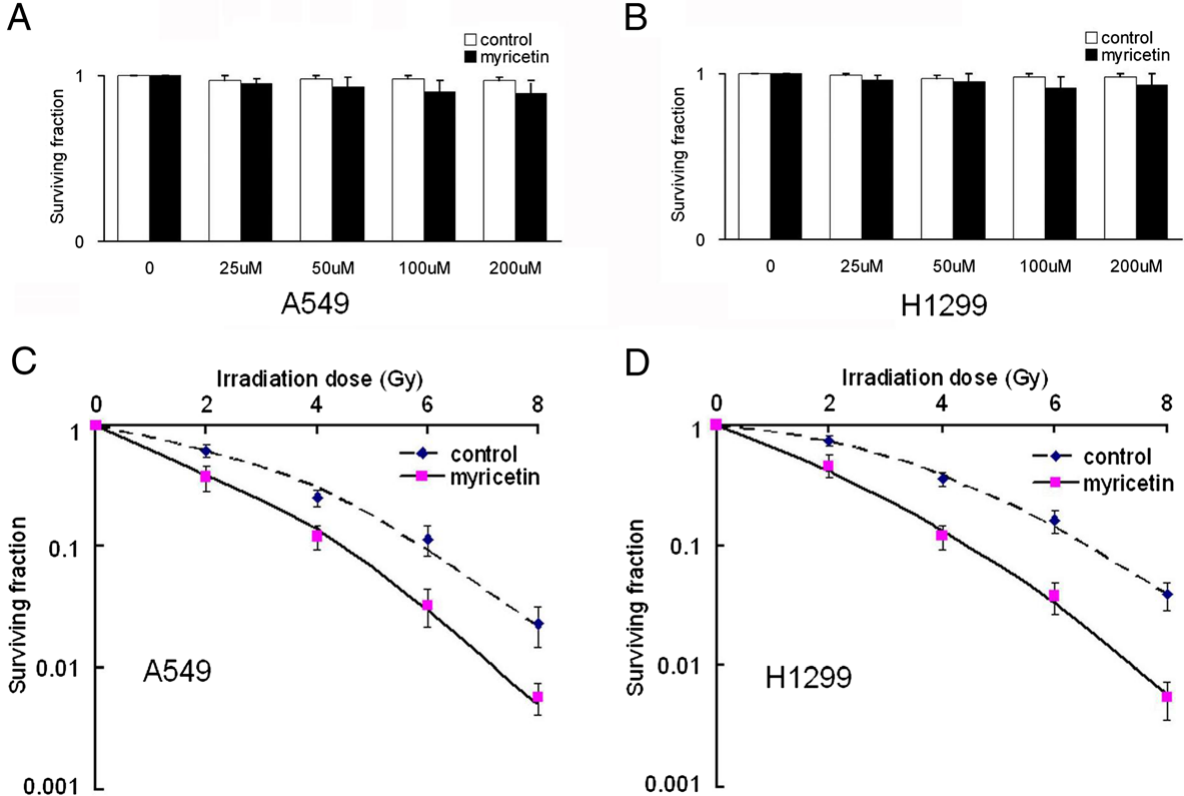
**Myricetin radiosensitizes cancer cells in vitro. (A)** and **(B)** Myricetin alone on clonogenic survival in A549 and H1299 cells lines. Cells were treated with indicated doses of myricetin for 24 h and colony formation assays were conducted. Compared to the control, there was little effect on clonogenic survival treated with different doses myricetin (*P* < 0.05). **(C)** Radiosensitivity was measured by colony formation assay in the human lung cancer cell line A549 treated with control or 25 μM of myricetin (1 h before IR and maintained for 24 h), Compared to the control, clonogenic survival of A549 cells treated with myricetin were decreased significantly under different irradiation doses. **(D)** Radiosensitivity of the human lung cancer cell line H1299. Shown are averages of triplicate samples. Standard errors are shown by error bars.

To determine the effects of myricetin on cells radiosensitivity, a clonogenic survival analysis was performed. It was found that treatment of the A549 cells with myricetin alone led to a minimal effect on clonogenic survival (Figure 1A and B). However, when combined with radiation, the surviving fraction decreased significantly (Figure 1C and D).

### Myricetin enhances radiosensitivity on inhibiting the A549 and H1299 cells’ proliferation

**Figure 2:**
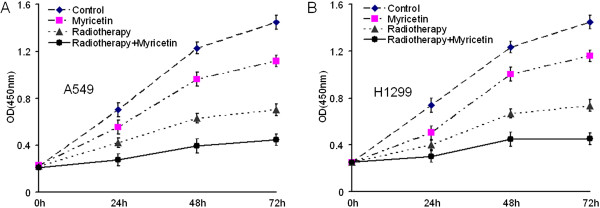
**Myricetin enhances radiosensitivity on inhibiting the tumor cell proliferation. (A)** Radiosensitivity was measured by CCK-8 assay in the human lung cancer cell line A549 treated with 25 μM of myricetin alone or myricetin plus radiotherapy (1 h before IR and maintained for 24 h), Compared to the radiotherapy alone group, the proliferation of A549 cells were slower down significantly in radiotherapy combine with myricetin group. **(B)** Radiosensitivity of the human lung cancer cell line H1299, which results was same as the A549.

CCK-8 assay was used to measure the effect of radiosensitization activity of myricetin on the growth and viability of A549 and H1299 cells in vitro. Compared to the control, both the proliferation of A549 cells (Figure 2A) and H1299 cells (Figure 2B) were inhibited significantly in myricetin group, radiotherapy group and radiotherapy + myricetin combination group. It was aslo found that myricetin-only had but only limited inhibitory effect on tumor cells proliferation. However, a significant decrease in tumor proliferation occurred for irradiated cells treated with myricetin. The result demonstrated that myricetin enhanced the radiosensitivity of cancer cells and has the function as a tumor radiosensitizer in lung cancer cells in vitro.

### Radiosensitizing effect of myricetin on tumor cells apoptosis

**Figure 3:**
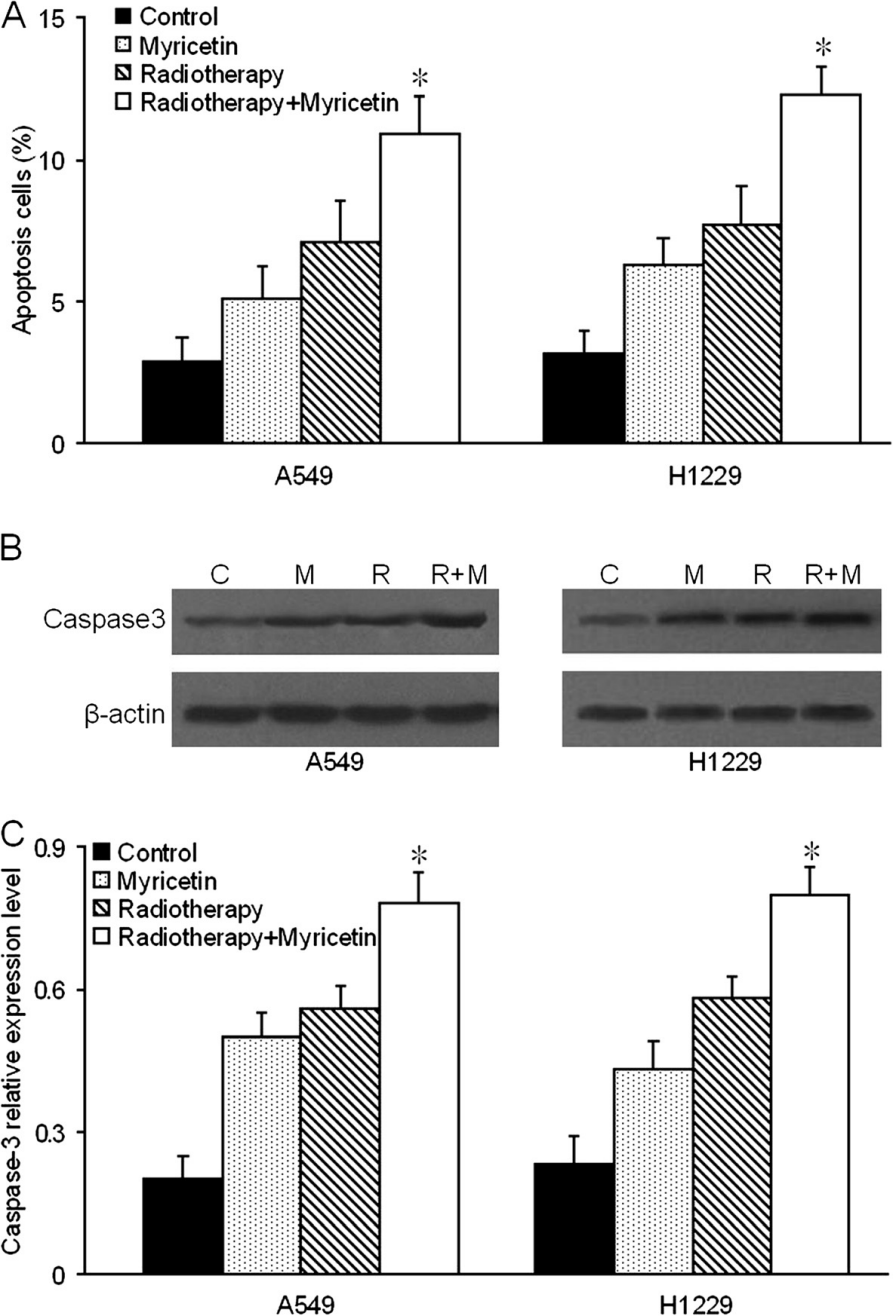
**Radiosensitizing effect of myricetin on tumor cells apoptosis. (A)** Myricetin increase the radiosensitivity of apoptosis rate in A549 and H1299 cells. The cells were stained by annexin V-FITC/PI, and cell apoptosis was analyzed by FCM. The data showed that apoptotic cells was statistically significant increased (*P < 0.05) in radiotherapy + myricetin combination group, compared to other three groups. **(B)** Western blot for Caspase-3 expression in control (c), myricetin-alone (M), radiotherapy-alone (R) and radiotherapy + myricetin combination (M + R) group. **(C)** Myricetin increase the Caspase-3 expression in occurred for radiotherapy in A549 and H1299 cells. The data aslo showed higher expression of Caspase-3 (*P < 0.05) in radiotherapy + myricetin combination group, compared to other three groups. Data are presented as the mean of triplicate experiments.

To investigate the apoptosis effect of myricetin-induced radiosensitization, we firstly used flow cytometry (FCM) to measure the effect of myricetin and irradiation on apoptosis in A549 and H1299 cells. As shown in Figure 3A, Compared to the control, both the apoptosis rate of A549 cells and H1299 cells were increased in myricetin-alone group and radiotherapy-alone group (*P* < 0.05). And furthermore, the apoptosis rate was significantly enhanced in radiotherapy + myricetin combination group. Meanwhile, compared with the radiotherapy alone group, the the apoptosis effect was obviously enhanced in radiotherapy + myricetin combination group (*P* < 0.05).

To further test the radiosensitizing effect of myricetin on tumor cells apoptosis, we aslo engaged the western blot to detect the expression of Caspase-3 protein. As shown in Figure 3B and C, compare to the control, the expression level of Caspase-3 were higher in myricetin-alone group and radiotherapy-alone group than in control group (*P* < 0.05). And the expression level of Caspase-3 were increased significantly in radiotherapy + myricetin combination group than in other three groups (*P* < 0.05).

### The radiosensitizing effect of myricetin in vivo

**Figure 4:**
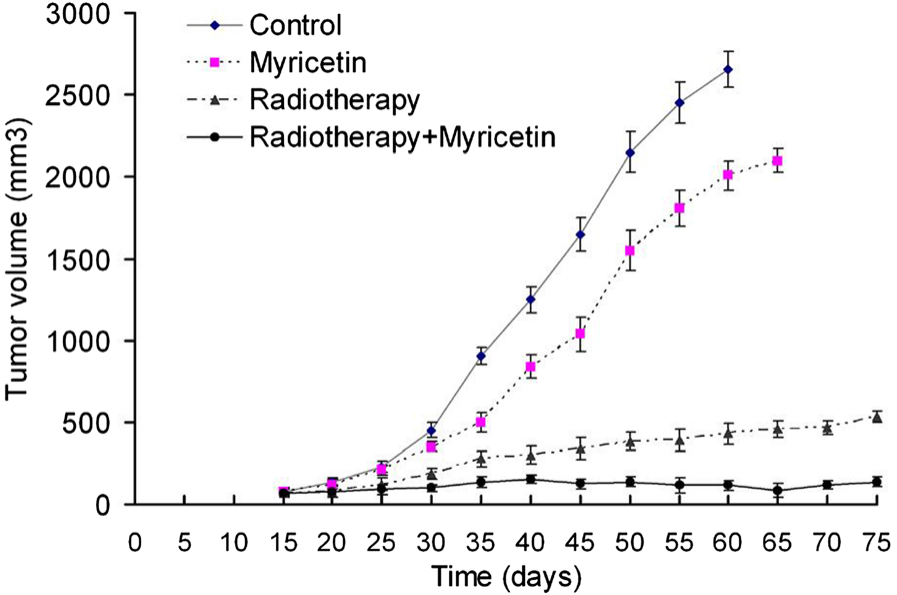
**A549 cell xenograft tumor for detecting myricetin sensitizing tumors to irradiation in vivo.** A549 cells were implanted subcutaneously in immunodeficient female BALB/C nude mice. Mice treated with myricetin alone (20 mg/kg, once daily for 14 days) or myricetin plus radiotherapy (myricetin was given 1 h before IR) at 2-Gy fractions for a total dose of 20 Gy. Shown are medium tumor volume of each group as function of time after implantation. The data curve showed that radiotherapy combine with myricetin could inhibit the tumor growth significantly, compared with other three groups.

Because myricetin has the radiosensitization activity on cell survival, prolifation and apoptosis in vitro, we performed a proof-of-principle experiment using a lung cancer A549 cell xenograft mice model to determine radiosensitizing effect of myricetin in vivo. The dose of myricetin used was 20 mg/kg (once daily for 12 days, for a total dose of 240 mg/kg). Radiation therapy (2 Gy/fraction, 5 times/week for a total dose of 20 Gy) was given locally to the tumor xenografts and scheduled to fit the myicetin administration. We found that myicetin-alone group had less inhibitory effect on tumor growth, compared with tumors treated with radiotherapy-alone (Figure 4). However, a significant slower down in tumor growth occurred for irradiated mice treated with myricetin. These results demonstrated that myricetin could increase lung tumor cell killed by radiation in vivo, and further indicated that myricetin can function as a powerful radiosensitizer for lung cancer.

## Discussion

Radiotherapy plays an important part in the management of lung cancer [24]. However, partial lung cancer cell’s resistance to radiotherapy affects therapeutic effects, and 5-year survival rate of radiotherapy alone is only 5%–10%, local recurrence occurs in 80% of patients, and metastasis occurs in 60% of patients [25]. Therefore, it is necessary to find a effective radiosensitizer to enhance tumor radiosensitivity, while with less negtive function to normal tissues.

Myricetin, a nontoxic dietary flavonoid, could be easily found in many natural foods and medicinal herbs [26,27]. Reports indicate that myricetin does not cause tumor formation in mice and attenuates the number of diol-epoxide-induced pulmonary tumors per mouse [28]. It inhibits polycyclic aromatic hydrocarbon metabolism and subsequent polycyclic aromatic hydrocarbon–DNA adduct formation in lung [29]. And many studies have shown that myricetin has antioxidant, anti-inflammatory and potent anticancer effects [15-18]. Therefore it was reasonable to hypothesize that myricetin might function as a radiosensitizer.

To test this hypothesis, we firstly conducted colony formation assays and prolifetation assay in lung cell lines (A549 and H1299), It was found that treatment of the cells with radiotherapy combined with myricetin could significantly decrease the surviving fraction and prolifetation of cancer cells. And in this study, we aslo engaged lung tumor bearing nude mice model to observe the radiosensitizing effect of myricetin in vivo. The result showed the tumor growth speed was significantly slower down in occurred for irradiated mice treated with myricetin. These data above demonstrate that myricetin has a radiosensitization potential in vitro and in vivo, and further indicated that myricetin can function as a powerful radiosensitizer for lung cancer.

Apoptosis is an important effect in the use of radiation to kill tumor cells, and it is now widely recognized that radiation-induced apoptosis may be used to measure the sensitivity of cells to radiation, with an increased rate of apoptosis meaning that the cells have a higher sensitivity to radiation [30-35]. In order to investigate the apoptosis effect of myricetin-induced radiosensitization, the apoptosis rate of A549 and H1299 cells were measured through FCM. The results showed that myricetin enhanced the radiosensitivity of apoptosis rate in lung cancer cells. And to further test the radiosensitizing effect of myricetin on tumor cells apoptosis, we detected the expression level of Caspase-3, which was plays a very important role during cells apoptosis. The data showed that myricetin could increase the expression of Caspase-3 in occurred for radiotherapy, which aslo indicated that myricetin could enhanced the radiosensitivity of apoptosis.

In conclusion, the results of the current study demonstrate the effect of myricetin on combination with radiotherapy enhances lung tumor radiosensitivity in vitro and in vivo, it could provied a novel insight into myricetin as a safe and potential agent for lung cancer radiosensitizers to enhance the effectiveness of radiotherapy.

## Abbreviations

IR: Irradiation; FCM: Flow cytometry; DDR: DNA damage responses; FBS: Fetal bovine serum.

## Competing interests

The authors declare that they have no competing interests.

## Authors’ contributions

SJZ, LW and GQZ designed the study, SJZ, LW GQZ and LM carried out the experiments and drafted the manuscript; HCL participated in the experiments and data analysis. All of the authors approved the final version of the manuscript.
